# Effect of Foliar Sodium Selenate and Nano Selenium Supply on Biochemical Characteristics, Essential Oil Accumulation and Mineral Composition of *Artemisia annua* L.

**DOI:** 10.3390/molecules27238246

**Published:** 2022-11-26

**Authors:** Lidia Logvinenko, Nadezhda Golubkina, Irina Fedotova, Maria Bogachuk, Mikhail Fedotov, Vladislav Kataev, Andrey Alpatov, Oksana Shevchuk, Gianluca Caruso

**Affiliations:** 1 Nikitsky Botanic Gardens, National Scientific Center of RAS, 298648 Yalta, Russia; 2 Analytical Laboratory Department, Federal Scientific Vegetable Center, 143072 Moscow, Russia; 3 Laboratory of Food Products, Institute of Nutrition and Biotechnology, 109240 Moscow, Russia; 4 Laboratory of New Metallurgical Processes and Alloys, A. Baikov Institute of Metallurgy and Material Science, 119334 Moscow, Russia; 5 Department of Agricultural Sciences, University of Naples Federico II, Portici, 80055 Naples, Italy

**Keywords:** antioxidants, minerals, oil yield and quality, selenium, solar activity, wormwood

## Abstract

Selenium (Se) biofortification of aromatic plants is a promising strategy to produce valuable functional food with high biological activity and enhanced essential oil yield. The experiment carried out in 2021 and 2022 on *A. annua* treated with sodium selenate or nano-Se sprayed on foliar apparatus demonstrated a significant increase in photosynthetic pigments, pectin, waxes, macro- and microelements and a decrease in malonic dialdehyde (MDA) accumulation. Contrary to literature reports, neither selenate nor nano-Se showed a beneficial effect on essential oil accumulation; the oil yield did not differ between the selenate treated and control plants but was halved by the nano-Se application. Extremely high variations in the number of essential oil components, as well as in the eucalyptol, artemisia ketone, camphor and germacrene D ratio in the 2021 and 2022 experiments were recorded. The analysis of the 2016–2022 data for oil yield and composition in the control plants revealed a direct correlation between the number of components and of solar flares, and a negative correlation between oil yield and the percentage of spotless days. Both control plants and plants fortified with selenium showed higher levels of germacrene D and lower levels of artemisia ketone in 2022, characterized by more remarkable solar activity compared to 2021. Nano-Se supply resulted in the highest percentage of germacrene D accumulation. The results of the present research highlight the importance of the solar activity effect on the essential oil yield and quality of aromatic plants.

## 1. Introduction

Biofortification of agricultural crops with Se is one of the most effective and economically beneficial methods for optimizing human Se status. The essentiality of this micro-element to mammals and the widespread Se deficiency in the world [1] have induced the development of functional food products with high levels of Se. Such products are highly valuable for immunity enhancement, antioxidant status improvement, the prevention of cancer, cardiovascular diseases, viral attack (including SARS-CoV-2), infertility and imbalanced brain activity [2,3]. Furthermore, utilization of Se derivatives in crop management provides an opportunity to increase yield and improve product quality [4,5,6,7]. In this respect, aromatic plants are of special interest, due to their ability to increase essential oil accumulation under Se supply. The information regarding the Se biofortification of aromatic plants is rather scant and refers to basil [7,8,9], cilantro [10], *Hypericum perforatum* [11,12] and borago plants [13]. In most cases, yield increase, essential oil improvement and polyphenol accumulation were recorded. *Artemisia annua* plants grown in the Moscow region under sodium selenate treatment revealed an antioxidant activity increase; although an insignificant increase in photosynthetic pigments accumulation and a low accumulation of essential oil [14].

*Artemisia* belongs to the Asteraceae family; it is relatively tolerant to high Se concentrations and demonstrates high antioxidant activity largely connected with high essential oil content [15]. Among the different plant families, Asteraceae is the richest one in Se-accumulators [15], which creates a lower risk of toxicosis under Se biofortification. Indeed, *A. edatifula* and *A. tridenrara* accumulated up to 60,000 µg Se kg^−1^ d.w. in the vicinity of the Redoubt Volcano eruption (Alaska), without growth inhibition [16], whereas according to our data, *A. vulgaris* may accumulate up to 1000 µg Se kg^−1^ d.w. in Kamchatka and only 38 µg Se kg^−1^ d.w in the Moscow region.

*A. annua* L. is included in the pharmacopeia of several countries (China, Vietnam, International WHO pharmacopeia) [17,18]. This plant is rich in antioxidants and demonstrates anti-tumor, anti-inflammatory, neuroprotective and analgesic effects, with a high activity against hepatitis B and other viruses, including SARS-CoV-2 [19,20,21,22]. In modern and traditional medicine, both wild and cultivated *Artemisia annua* L. plants have been used [23]. The high adaptability of this species results in the worldwide diffusion of this species and its industrial cultivation in several countries, such as China, India, Vietnam, Kongo, Brazil, Kenia, and Tanzania [17]. *A. annua* is rich in flavonoids, phenolic acids, tannins, saponins, phytosterols, and essential oil, containing artemisia ketone, camphor and germacrene D. This plant is a unique source of artemisinin, used in malaria treatment [24,25]. The comparison of the mentioned properties of *A. annua* components with the biological activity of Se compounds raises high prospects for plant Se biofortification. However, the efficiency of Se treatment is greatly affected by the chemical form used: selenates, selenites, organic forms, and nano-Se. Among the latter, Se nanoparticles are considered the least toxic, while the selenate form is the most mobile one [26,27]. Limited information about the effect of Se forms on *Artemisia* plant growth and development suggests the necessity to evaluate different biofortification approaches in *A. annua* Se supply. Taking into account that *A. annua* shows optimal growth and essential oil accumulation under rather high values of mean annual temperatures (10–15 °C) and solar radiation [28,29], the southern world regions are the most attractive. 

The aim of the present investigation was the comparative evaluation of foliar Se biofortification of *A. annua* in Crimean southern coast conditions, using two forms of this microelement: sodium selenate and nano-Se particles.

## 2. Results and Discussion

### 2.1. Morphological Characteristics and Yield

**Figure 1:**
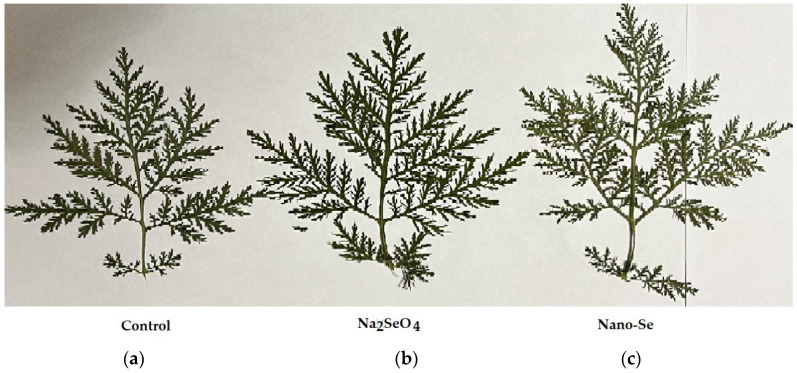
*A. annua* leaves without Se supply (**a**) and biofortified with sodium selenate (**b**) and nano-Se (**c**).

The southern Crimean Sea coast is characterized by a dry subtropical climate and carbonate, light clay soils. In these conditions, plant productivity is of great priority. In the Nikitsky Botanic Gardens collection of *Artemisia* species, *A. annua* has a high content of P and K, low level of Na [15], and essential oil accumulation as much as 0.5% f.w. [30]. Our experiment, based on Se biofortification of *A. annua,* has revealed the beneficial effect of Se supply on plant growth and development, both as sodium selenate and nano-Se application. Despite only a tendential increase in plant height, leaf width and length being recorded (Table 1), Se fortified plants showed stronger leaves compared to the control (Figure 1), which was in accordance with the corresponding median values of these parameters (Table 1).

### 2.2. Photosynthetic Pigments

The beneficial effect of Se on plant growth and development is closely related to the increase in photosynthetic pigment production [31]. Table 2 data demonstrate a remarkable increase in chlorophyll a, chlorophyll b and carotene accumulation in *A. annua* leaves. Indeed, compared to the control, sodium selenate resulted in a 1.81, 1.85 and 1.76 increase in chlorophyll a, chlorophyll b and carotene, respectively; while the application of nano-Se resulted in a 2.13 (chlorophyll a), 2.46 (chlorophyll b) and 1.48 (carotene) increase. The total chlorophyll content increased by 1.82 (sodium selenate) and 2.29 (nano-Se) times due to Se supplementation. 

The data obtained indicate one more peculiarity of the nano-Se effect (Table 2), i.e., the total chlorophyll to carotene ratio was the highest in the plant leaves subjected to nano-Se supply. Carotenoids have a major function in protecting chlorophyll and the surrounding cells from light damage. Chlorophylls often generate toxic reactive oxygen species, which cause diverse cellular damage, and they are particularly prone to generating such free radicals under high light conditions. Carotenoids are able to absorb excess light, diverting it from chlorophyll. Unlike chlorophyll, carotenoids can harmlessly convert excess excitation energy to heat [32].

Selenium can contribute by enhancing the quantum efficiency of photosystem II in plants [33]. The yield increase of potato plants treated with Se indicated that this microelement presumably had the effect of allocating more photosynthesis products to the tubers [31]. In a hydroponic experiment regarding sodium selenite treatment in *Brassica,* the recorded 43% increase in seed production was attributed to the higher total respiratory activity of plants [34].

### 2.3. Antioxidant Status

Despite the remarkable differences in photosynthetic pigment accumulation between the control and Se treated plants recorded in the present work, no significant differences were detected for AOA and TP (Table 3). The latter fact may reflect the lack of significant oxidative stress in *A. annua* plants during growth and development. 

**Figure 2:**
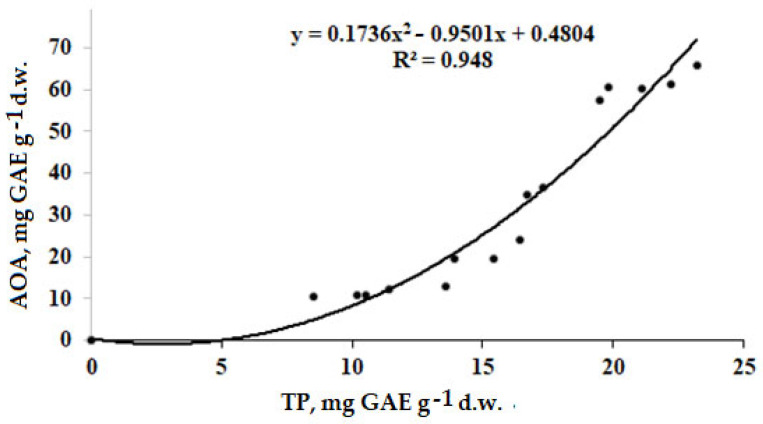
Correlation between AOA and TP in *A. annua* control plants and plants supplied with selenate (+6) and nano-Se (r = 0.974, *p* < 0.0001).

Notably, within the total AOA, the percentage of polyphenols was the highest in all plant parts, showing an increasing trend from leaves (34.7–33.5%) to stems (55.4–56.3%) and roots (94.1–93.9%). Indeed, this phenomenon suggests the predominant role of polyphenols (TP) in the antioxidant status of *A. annua* roots, but the remarkable participation of the essential oil in the corresponding activity of leaves [15]. The high correlation coefficient of 0.974 detected between TP and AOA in our research (Figure 2) is close to that recorded in *A. annua* plants grown in the Moscow region [14].

The importance of polyphenol participation in plant antioxidant defense is well documented [35], and the close relationship between TP and AOA has been described in various agricultural crops [36] and tree bark [37], indicating the general trend of this phenomenon.

Proline, as a stabilizer of sub-cellular structures and a natural antioxidant, and malonic dialdehyde, as an indicator of lipid peroxidation, are frequently used to characterize the stress intensity and antioxidant defense levels in plants [38]. The data presented in Table 3 indicate only slight changes of proline accumulation in *A. annua* leaves as a result of Se supply, and a small decrease in the MDA level in plants treated with nano-Se. These data indirectly confirm the low intensity of environmental stresses in the conditions of this experiment.

Among the different plant protection patterns against environmental stresses, cuticle wax is known to provide powerful protection against UV-radiation, air pollutants, pathogens, and herbivory [39]. The data presented in Table 3 indicate that both sodium selenate and nano-Se are able to increase wax concentration in *A. annua*. To date, no information has been obtained proving the beneficial effect of Se on wax accumulation in plants. However, to further confirm the similarity of several crops with reference to this phenomenon, future investigations on plants biofortified with Se are needed to unveil other details of the connected mechanisms and the factors affecting the Se beneficial effect.

### 2.4. Essential Oil

The importance of essential oil in plants’ antioxidant defense and the high biological activity of *A. annua* oil, capable of inducing apoptosis in hepatocarcinoma cells [40], indicate the significance of these compounds’ accumulation. Many attempts have been made to indicate the factors affecting essential oil accumulation in *A. annua* leaves. Essential oil yield and chemical composition were shown to depend greatly on plants’ habitat, climate [41], the presence of AMF in soil [30], planting date [42], plant ontogenetic conditions [43,44], and temperature [45]. Though remarkable variations of yield and composition have been recorded in different world regions [41], *A. annua* oil is rich in mono- and sesquiterpenes and includes major constituents, such as camphor, artemisia ketone, eucalyptol and germacrene D. However, to date, the wide range in values of *A. annua* oil parameters have received poor explanations. 

The data presented in Table 4 indicate the lack of statistically significant changes in essential oil accumulation between the control and sodium selenate treated plants and the 2-fold decrease in this parameter due to nano-Se supply. The calculation of the essential oil yield per whole plant provided a more optimistic outcome: 1.76 ± 0.17 g in control; 1.96 ± 0.19 g in Se^+6^ and 1.92 ± 0.13 g in the nano-Se group with medians of 1.74, 1.99 and 1.96 g, respectively, though the differences were not statistically significant. The narrow range of beneficial Se concentrations necessary for the improvement of essential oil accumulation in aromatic plants and significant differences in the response to various Se derivatives supply [9,10,11,12,13] may partially explain the results of the present investigation.

**Figure 3:**
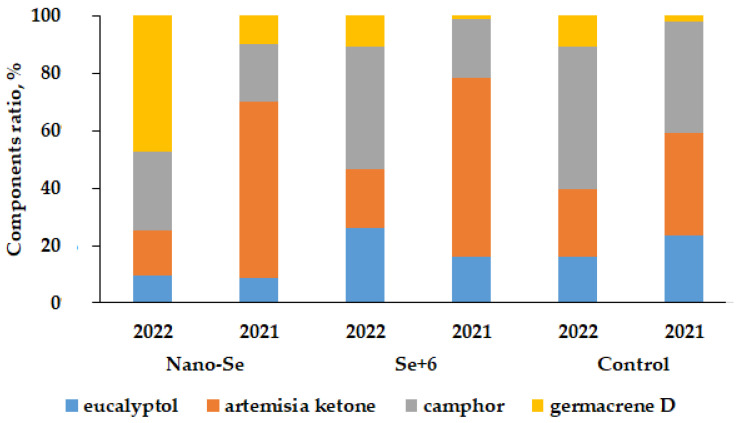
The main components ratio in *A. annua* essential oil without and under Se supply in 2021–2022.

Furthermore, a higher number of *A. annua* oil components in the control plants and plants subjected to sodium selenate and nano-Se supply were recorded in 2022 compared to the previous year (Table 4): 75 versus 54 for control plants, and 64 versus 49 for selenate treated plants, with the highest components level detected in nano-Se treated plants in 2022. Table 4 and Figure 3 data indicate the extremely high variations in the main oil components content in 2021 and 2022. Indeed, eucalyptol content varied from 2.11 to 15.58%, artemisia ketone from 3.97 to 42.55%, camphor from 5.01 to 27.65%, and germacrene D concentration from 0.93 to 12.20%. The control and Se treated plants in 2021 were characterized by a significantly higher content of artemisia ketone, among the main oil components, compared to the 2022 data (Figure 3), despite similar growth conditions and treatments; on the contrary, the 2022 data showed the predominance of germacrene D (Figure 3). The highest increase in germacrene D percentage was recorded in nano-Se supplied plants. The main functional group of camphor and artemisia ketone is the ‘C=O’ group, while eucalyptol is a cyclic ether and germacrene D is an acyclic unsaturated hydrocarbon with no functional groups. Notably, the results indicate that climate peculiarities in 2022 and the nano form of Se elicited the increase in biosynthesis of germacrene D.

The detected great variability of *A. annua* essential oil composition does not presumably affect its biological activity to any great extent, such as its antimicrobial properties, due to the well-known synergism between them [41]. 

The two research years, 2021 and 2022, were characterized by significant differences both in precipitation levels from June to September, and in solar activity, which showed a great influence on *A. annua* essential oil. Indeed, the number of solar spots increased from 30 (in 2021) to 77 (in 2022), while the number of solar flares increased from 398 to 1530.

**Figure 4:**
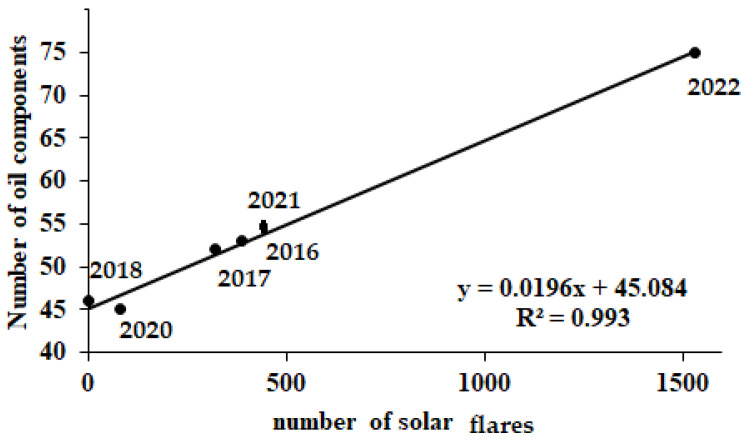
Number of oil components in *A. annua* leaves from 2016 to 2022 (r = 0.996; *p* < 0.0001).

**Figure 5:**
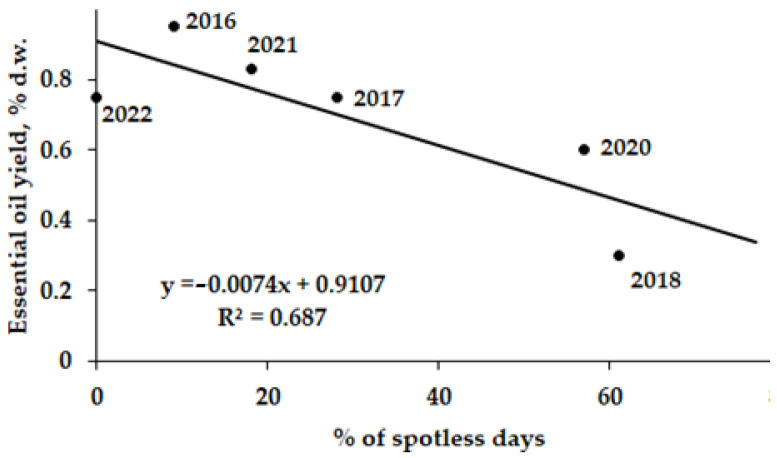
Accumulation of essential oil in *A. annua* leaves from 2016 to 2022 (r = −0.829; *p* < 0.01).

To indicate the role of solar activity on essential oil accumulation in *A. annua* leaves, a comparison was made between the solar activity parameters, oil yield, and the number of oil components in control plants grown from 2016 to 2022 at the experimental field of Nikitsky Botanic Gardens. The results revealed a significant positive correlation between the number of oil components and of solar flares (Figure 4), and a negative correlation between the essential oil yield and the percentage of spotless days (Figure 5).

The high level of probability of the above correlations may be connected to the fact that *A. annua* completes its cycle in two seasons of the same year and the essential oil is not biosynthesized in its plant parts to play the function of a reserve substance.

The non-significant correlations between artemisia ketone, eucalyptol, germacrene D and camphor contents with the solar activity parameters raises the need to further monitor the essential oil accumulation and deepen the investigation into the related phenomena. 

### 2.5. Polysaccharides

The increased photosynthetic products of the Se-treated plants may have stemmed from the augmented number of photoreceptors and, accordingly, by a higher synthesis of carbohydrates beneficial to plant growth [31]. Table 5 data indicate that both sodium selenate and nano-Se treatment significantly increased the pectin and nitrate contents as well as the Total Dissolved Solids (TDS). Particularly, the pectin concentration increased by 67% and 23% upon the Se^+6^ and nano-Se supply, respectively. Non-soluble, soluble and total fiber were not significantly affected by the selenium treatments (Table 5).

Water-soluble polysaccharides of *Artemisia* species demonstrate a wide variety of biological properties including immunomodulating, antitumor and anticomplement activities, and the inhibition of HepG2 cell growth [46]. The investigations of Huo et al. [46] reported that increased galacturonic acid content in *A. annua* leaves is highly valuable in *A. annua* anticomplement activity and heat-clearing effect. According to Brisibe et al. [47] *A. annua* leaves accumulate the highest levels of fiber and ash. Table 5 data show that Se supply both in the form of sodium selenate and nano-Se increased the accumulation of nitrates slightly and of the total dissolved solids significantly. The latter parameter reflects the content of water soluble polysaccharides, protein and minerals.

### 2.6. Elemental Composition

The evaluation of *A. annua* mineral composition revealed a significant accumulation of all the elements tested as a consequence of Se supply. Indeed, among the macroelements Mg, K and P are known to increase the content of chlorophyll a and the sum of chlorophyll (a + b). Being a central atom in the chlorophyll molecule, Mg stimulates the formation of chlorophyll affecting the efficiency of photosynthesis, CO_2_ fixation and assimilate transport [48,49]. According to Zielewicz et al. [49], Mg and P promote the accumulation of chlorophyll pigments in mountain melick plants and the decrease in carotene content. The obtained data (Table 6) show that a significant increase in Mg and K content in *A annua* leaves due to Se supply (both in ionic and nano-form) is in accordance with the higher photosynthetic activity (Table 2) and biomass (Table 1) of biofortified plants. 

**Figure 6:**
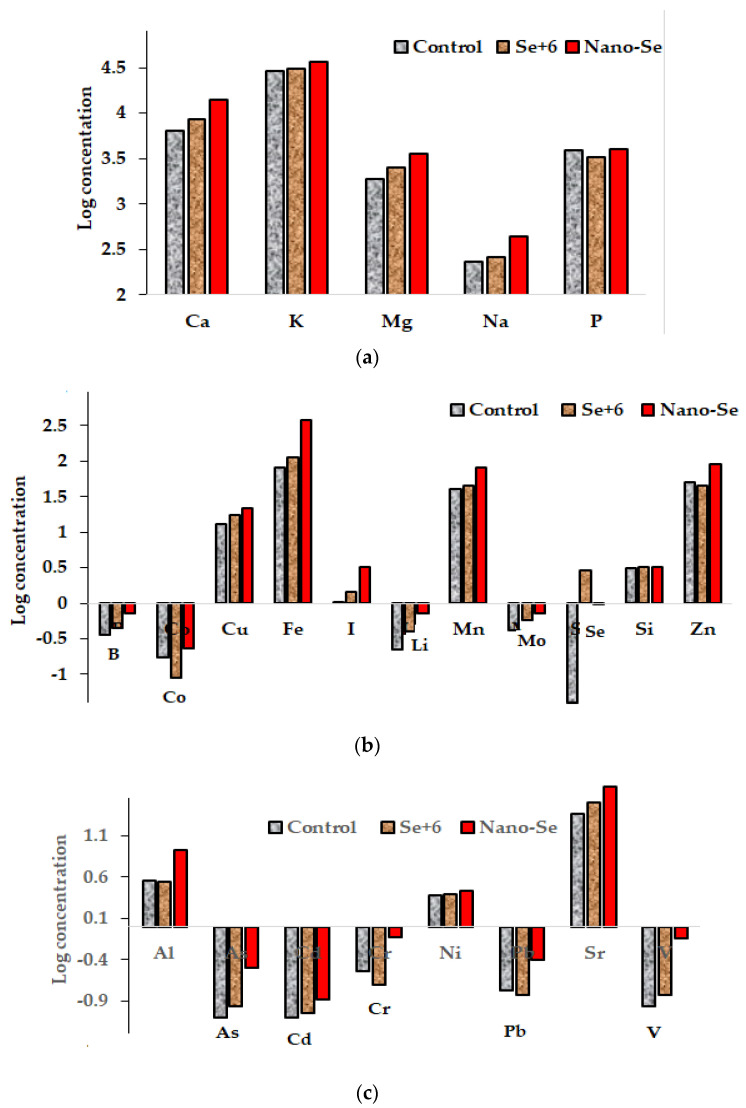
Effect of Se foliar biofortification on mineral composition of *A. annua* leaves: (**a**) macroelements; (**b**) microelements; (**c**) Al, As and heavy metals.

Se-nanoparticles showed the highest beneficial effect on microelement accumulation, except for Se itself, which is known to be more bioavailable in the form of sodium selenate than nano-Se [50]; and Si, whose concentration did not differ between the control and the Se-treated plants. A higher iodine accumulation due to nano-Se supply, compared to sodium selenate application, is of special interest to produce functional food with a valuable content of both elements, the latter are known to exhibit a similar metabolic activity in humans [51]. To date, no information relevant to the effect of Se nanoparticles on the iodine accumulation in plants has been published. The macro- and microelements increase recorded in this research, due to nano-Se supplementation in plants, indicates a significant nutritional status improvement. From a practical point of view, the chance to enhance the I, Fe, Mn and Zn contents by nano-Se supply, compared to sodium selenate application, has raised an important perspective (Figure 6).

Moreover, these results indicate that Se supply (both as sodium selenate and nano-Se) in normal plant growing conditions, i.e., without significant heavy metals uptake, may increase the concentration of heavy metals, Al and even As within a safe concentration range (Table 6, Figure 6). These results contradict the well-known protective effect of all forms of Se against heavy metals [52,53]. As can be seen in Table 6, within the conditions of our research, the nano-Se form stimulated the accumulation of all analyzed elements except Ni, while sodium selenate decreased only the concentration of Cr, with no effect on Al, As, Cd, Ni and Pb content. The anomalous Se and heavy metals interaction is typical of a low toxic element concentration in the environment and has already been described for other agricultural crops. Notably, significant increases in Sr and V were recorded in Mitsuba under sodium selenate foliar application [54], Al, Cr, Ni and V in *A. ursinum* leaves [55], and Al, As, Pb and V in Indian mustard [56]. Further investigations will be useful to reveal the mechanism of the mentioned interactions and highlight the most important peculiarities of these phenomena.

## 3. Materials and Methods

### 3.1. Growing Conditions and Experimental Protocol 

The research was conducted on *A. annua*, cultivar Novichok, in 2021 and 2022, from April to October at the experimental open field of Nikitsky Botanic Gardens, situated by the shore of the Black Sea (44°31′ N., 34°15′ E, 200 m above sea level), characterized by a Mediterranean-type dry subtropical climate, with a mean year temperature of 13.5 ± 1.5 °C and an average daily temperature above 5 °C from the beginning of March to the end of October (Table 7). The experiment was carried out on an agro-brown, slightly carbonate, light clay soil with 3.0% humus, 5.4% carbonates and a pH of 7.8. The annual precipitation was 560 to 619 mm with typical predominance in the winter–spring period.

Sowing was performed from 20 to 30 March. The plants were supplied with Se thrice at 20-day intervals before the budding stage, from June to August, by foliar treatment with 0.39 mM selenium solutions: sodium selenate (73.2 mg L^−1^) and a colloidal solution of nano-Se (30.6 mg L^−1^). The control plants were sprayed with water. Three replicates were set up in this study, with each plot covering 1 m^2^. To exclude the interference of other factors, no fertilizers were applied during the experiment. The results were expressed as means of the two-year data both for the control and selenium supplied plants. The morphological determinations were performed on 10 plants per replicate.

### 3.2. Preparation and Characterization of Selenium Colloidal Solution

Selenium nanoparticles were obtained by pulse laser ablation in deionized water. The solid target of pure gray Se was immersed at the bottom of a static glass cell with 100 mL of deionized water. The target was irradiated with a laser beam of nanosecond Nd:YAG laser. The laser wavelength was 1064 nm, pulse duration was 12 ns, pulse frequency was 1 Hz, and average pulse energy was 2.5 J. The laser beam was focused on the target via lens. Depending on the time of irradiation and energy density, it is possible to obtain a Se colloidal solution with specified concentration and particle size distribution.

The Se nanoparticles’ concentration was measured by inductively coupled plasma atomic emission spectrometry (ICP-AES) with an ULTIMA 2 (Horiba Jobin Yvon, Longjumeau, France) spectrometer. The hydrodynamic size and zeta potential (ZP) of the Se nanoparticles were characterized by the dynamic light scattering (DLS) method using Photocor Compact Z (Photocor, Tallin, Estonia) laser analyzer with a wavelength λ = 589 nm and laser rated-power output of 32 mW at 25 °C. The morphology of the nanoparticles was studied by electron energy loss spectroscopy using a JEM-2100 (JEOL, Akishima, Japan) transmission electron microscope.

**Figure 7:**
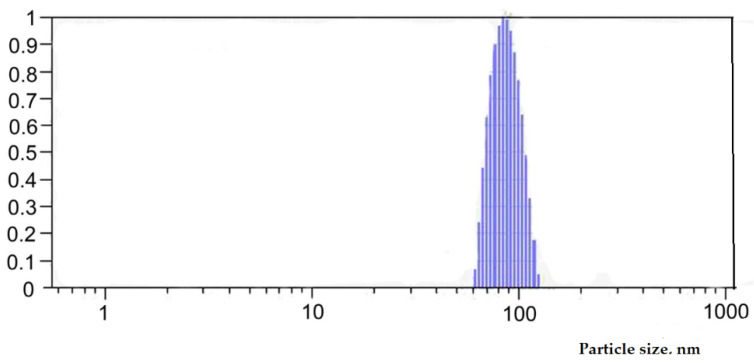
Selenium particle size distribution of colloidal solution.

The concentration of Se in the colloidal solution was equal to 30.6 mg L^−1^. Figure 7 shows the particle size distribution in the colloidal solution. It was shown that the obtained nanoparticles had monomodal size distribution with an average particle size of about 100 nm. The ζ-potential value of the colloidal solution was −39.7 mV, which indicated a significant stability of the solution.

**Figure 8:**
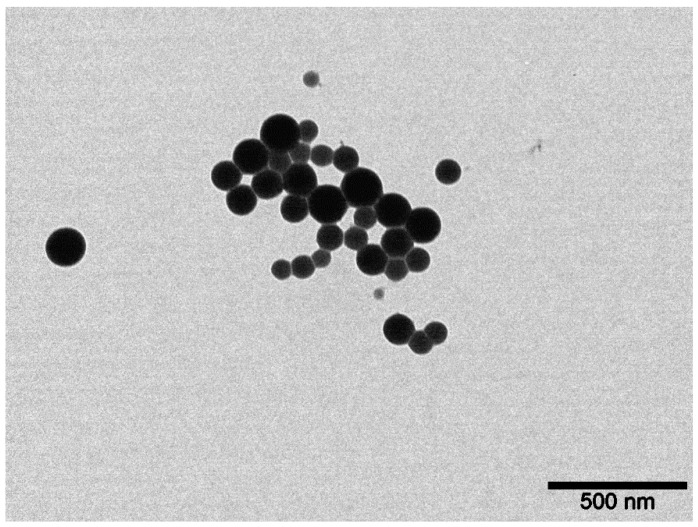
TEM image of spherical shaped Se nanoparticles in colloidal solution.

Figure 8 reveals the spherical shape of the particles with an average diameter of about 100 nm. These data are in agreement with the DLS results shown in Figure 7.

### 3.3. Sample Preparation

After harvesting and removing the soil particles from the roots and stems, the two latter plant parts were separated from each other, washed with water, dried with filter paper, and then individually weighed; the same procedure was followed for the leaves. The samples were homogenized and fresh homogenates were used for the determination of nitrates, while leaf homogenates were used only for the determination of photosynthetic pigments concentration. Sample aliquots of the roots, stems and leaves were dried at 70 ℃ to constant weight and used for the determination of total polyphenols content (TP), total antioxidant activity (AOA), polysaccharides (PS), wax and Se. 

### 3.4. Biochemical and Elemental Composition Analyses

#### 3.4.1. Nitrates

Nitrates were assessed using an ion-selective electrode on ionomer Expert-001 (Econix Inc., Moscow, Russia). Five grams of fresh *A. annua* leaves were homogenized with 50 mL of distilled water. Forty-five ml of the resulting extract were mixed with 5 mL of 0.5 M potassium sulfate background solution (necessary to adjust the ionic strength) and analyzed through an ionomer for nitrate determination.

#### 3.4.2. Wax

The wax content in the *A. annua* leaves were assessed spectrophotometrically by Unico 2804 UV spectrophotometer (Dayton, NJ, USA), using the absorption value of *A. annua* hexane extracts at 260 nm [25]. As an external standard, 0.02% paraffin solution in hexane was used. The results were expressed as mg of paraffin equivalent per g of dry weight (mg PE g^−1^ d.w).

#### 3.4.3. Photosynthetic Pigments

The photosynthetic pigments were measured using 96% ethanolic extracts of *A. annua* leaves according to Lichtenthaler [57].

#### 3.4.4. Dietary Fiber

The dietary fiber content was assessed gravimetrically after enzymatic hydrolysis and the removal of protein and starch according to [58].

#### 3.4.5. Pectin

The pectin content was evaluated gravimetrically after extraction with 0.05 M hydrochloric acid in a water bath and ethanol precipitation [59].

#### 3.4.6. Total Polyphenols (TP)

The total polyphenols were determined in a 70% ethanol extract using the Folin–Ciocalteu colorimetric method as previously described [60]. One gram of dry *A. annua* homogenates was extracted with 20 mL of 70% ethanol at 80 ℃ for 1 h. The mixture was cooled down and quantitatively transferred to a volumetric flask, and the volume was adjusted to 25 mL. The mixture was filtered through filter paper, and 1 mL of the resulting solution was transferred to a 25 mL volumetric flask, to which 2.5 mL of saturated Na_2_CO_3_ solution and 0.25 mL of diluted (1:1) Folin–Ciocalteu reagent were added. The volume was brought to 25 mL with distilled water. One hour later the solutions were analyzed through a spectrophotometer (Unico 2804 UV, Dayton, NJ, USA), and the concentration of polyphenols was calculated according to the reaction mixture absorption at 730 nm. As an external standard, 0.02% gallic acid was used. The results were expressed as mg of gallic acid equivalent per g of dry weight (mg GAE g^−1^ d.w).

#### 3.4.7. Antioxidant Activity (AOA) 

The antioxidant activity of *A. annua* roots, stems and leaves was assessed using a redox titration method [60] via titration of 0.01 N KMnO_4_ solution with ethanolic extracts of dry samples, produced as described in Section 3.4.6. The reduction in KMnO_4_ to colorless Mn^+2^ in this process reflects the quantity of antioxidants dissolvable in 70% ethanol. The values were expressed in mg gallic acid equivalents per g of dry weight (mg GAE g^−1^ d.w.).

#### 3.4.8. Extraction and Analysis of the Essential Oil 

The essential oil content in the aerial plant parts was determined during the full flowering stage. For this purpose, 50 g of each dry sample were hydro-distilled in a Ginsberg-type apparatus for 2 h and then the percentage and yield of essential oils were calculated [61]. The essential oils were dried over anhydrous sodium sulfate, stored in dark glass vials, and kept at 4 °C. The composition of essential oil was investigated by gas-chromatograph Chromatec-Kristall 5000.2 (Chromatec Inc., Ioshkar-Ola, Russia) with a mass-spectrographic detector. Volatile components were separated on a capillary column CR-5 ms (5% phenylmethyl-polysiloxane, 0.25 mm × 30 m; 0.25 µm film thicknesses). The temperature of the injector and transfer line were set to 250 °C and 300 °C, respectively. The oven was heated up to 75 °C, with a subsequent increase of 4.0 °C min^−1^, up to 240 °C; the evaporator temperature was 250 °C. The following conditions were adopted: split ratio 1:25, at 1.1 mL min^−1^ flow, with helium as carrier gas, and injection volume of 1 mL of essential oil diluted in dichloromethane (1:300 *v*/*v*). The components of the essential oils were identified by a comparison of their retention indices relative to (C_8_–C_30_) n-alkanes (Sigma-Aldrich, Buchs, Switzerland) and Supelco analytical standards (St Louis, MO, USA) and via comparison of their mass-spectra with those of the NIST 14 mass spectra collection (National Institute of Standards and Technologies, Gaithersburg, MD, USA) [62].

#### 3.4.9. Elemental Composition

Aluminum, As, B, Ca, Cd, Co, Cr, Cu, Fe, Hg, K, Li, Mg, Mn, Na, Ni, P, Pb, Si, Sn, Sr, V, and Zn contents in dried homogenized samples were assessed using ICP-MS on a quadruple mass-spectrometer Nexion 300D (Perkin Elmer Inc., Shelton, CT, USA), equipped with the seven-port FAST valve and ESI SC DX4 autosampler (Elemental Scientific Inc., Omaha, NE, USA) at the Biotic Medicine Center (Moscow, Russia). Rhodium 103 Rh was used as an internal standard to eliminate instability during the measurements. Quantitation was performed using an external standard (Merck IV, multi-element standard solution); Perkin-Elmer standard solutions for P, Si, and V, and all the standard curves were obtained at five different concentrations. For quality control purposes, internal controls and reference materials were tested together with the samples daily. Sample microwave digestion was carried out using sub-boiled HNO_3_ (Fluka No. 02, 650 Sigma-Aldrich, Co., Saint Louis, MO, USA) diluted 1:150 with distilled deionized water in the Berghof SW-4 DAP-40 microwave system (Berghof Products + Instruments GmbH, 72, 800 Eningen, Germany). The instrument conditions and acquisition parameters were: plasma power and argon flow, 1500 and 18 L min^−1^, respectively; aux argon flow, 1.6 L min^−1^; nebulizer argon flow, 0.98 L min^−1^; sample introduction system, ESI ST PFA concentric nebulizer and ESI PFA cyclonic spray chamber (Elemental Scientific Inc., Omaha, NE, USA); sampler and slimmer cone material, platinum; injector, ESI Quartz 2.0 mm I.D.; sample flow, 637 L min^−1^; internal standard flow, 84 L min^−1^; dwell time and acquisition mode, 10–100 ms and peak hopping for all analytes; sweeps per reading, 1; reading per replicate, 10; replicate number, 3; DRC mode, 0.55 mL min^−1^ ammonia (294993-Aldrich Sigma-Aldrich, Co., St. Louis, MO 63103, USA) for Ca, K, Na, Fe, Cr, V, optimized individually for RPa and RPq; STD mode, for the rest of analytes at RPa = 0 and RPq = 0.25. 

Trace levels of Hg and Sn in the samples were not considered and, accordingly, they were not included in the Tables.

#### 3.4.10. Determination of Selenium

Selenium was analyzed using the fluorimetric method previously described for tissues and biological fluids [63]. Dried homogenized samples were digested via heating with a mixture of nitric and perchloric acids, subsequent reduction in selenate (Se^+6^) to selenite (Se^+4^) with a solution of 6 N HCl, and formation of a complex between Se^+4^ and 2,3-diaminonaphtalene. The calculation of the Se concentration was performed by recording the piazoselenol fluorescence value in hexane at 519 nm λ emission and 376 nm λ excitation. Each determination was performed in triplicate. The precision of the results was verified using a reference standard for Se fortified chervil stem powder in each determination, with a Se concentration of 1865 μg· Kg^−1^ (Federal Scientific Vegetable Center).

#### 3.4.11. Proline

The proline concentration was determined according to Ábrahám [64] with slight modification. Fifty mg of dry homogenized *Artemisia* leaves were homogenized with 10 mL of 3% sulfur salicylic acid in a mortar. The mixture was filtered and 1 mL of the resulting filtrate, 2 mL of ninhydrin reagent, and 2 mL of acetic acid were heated at 95 °C for 1 h. The proline concentration was assessed using the absorption value of the reaction mixture at 505 nm and a calibration curve with 5 different proline (Merck) concentrations.

#### 3.4.12. Malonic Dialdehyde

Dried homogenized leaves of *A. annua* (100 mg) were heated at 95 °C for 30 min with 5 mL of 0.5% tiobarbituric acid containing 10% trichloroacetic acid. The resulting solutions, after cooling, were filtered and the absorption at 532 nm was determined. The calculation of the MDA concentration was performed using the extinction value equal to 155 [65].

### 3.5. Statistical Analysis

The data were processed by an analysis of variance and mean separations were performed through the Duncan’s multiple range test, with a reference to 0.05 probability level, using SPSS software version 21 (Armonk, NY, USA). Data expressed as percentages were subjected to angular transformation before processing.

## 4. Conclusions

According to the obtained results, sodium selenate and nano-Se supply provide a beneficial effect on *A. annua* growth and development, improving photosynthetic pigments, pectin, and wax, as well as macro- and trace elements accumulation. A positive correlation between the number of essential oil components and solar activity, and a negative correlation between essential oil yield and the number of days free of solar spots indicate a significant cosmic effect on plant productivity. Further investigations are desirable to reveal the mechanism of such interactions and produce a model to predict the essential oil accumulation in plants.

## Figures and Tables

**Table 1:** Morphological characteristics of *A. annua*.

Parameter		Control	Se^+6^	Nano-Se
Plant height (cm)	M ± SD *	176.7 ± 6.2 a	180.6 ± 3.5 a	184.8 ± 5.6 a
	Range	170–185	175–185	177–193
	Median	177.0	181.0	186.5
Plant diameter (cm)	M ± SD *	65.1 ± 2.8 b	71.4 ± 3.6 ab	73.7 ± 3.2 a
	Range	60–69	67–77	67–77
	Median	65.0	71.5	74.5
Leaf length (cm)	M ± SD *	11.8 ± 0.4 b	12.5 ± 0.3 ab	12.9 ± 0.5 a
	Range	11.5–12.1’	12.0–12.8	12.1–13.8
	Median	11.9	12.5	12.9
Leaf width (cm)	M ± SD *	11.1 ± 0.4 a	11.8 ± 0.2 a	11.7 ± 0.2 a
	Range	10.6–11.8	11.5–12.0	11.5–12.0
	Median	11.1	11.9	11.7
Petiole length (cm)	M ± SD	91.8 ± 5.0 a	91.9 ± 2.6 a	89.4 ± 3.2 a
	CV (%)	5.4	2.8	3.6
	Range	83–98	88–96	85–94
	Median	92.5	92.5	89.5
Plant biomass (Yield) (kg m^−2^)	M ± SD	320.0 ± 33.7 a	333.9 ± 30.8 a	338.0 ± 26.2 a
	CV (%)	10.5	9.2	7.8
	Range	291.7–398.0	296.3–374.0	290.7–358.6
	Median	307.9	333.4	341.0

* mean of 10 plants. Along each line, the values with the same letters do not differ statistically according to Duncan’s test at *p* < 0.05.

**Table 2:** Photosynthetic pigments of *A. annua* leaves.

Parameter	Control	Se^+6^	Nano-Se
Chlorophyll a (mg g^−1^ f.w.)	2.39 ± 0.20 b	4.32 ± 0.42 a	5.09 ± 0.48 a
Chlorophyll b (mg g^−1^ f.w.)	1.60 ± 0.14 c	2.96 ± 0.30 b	3.93 ± 0.40 a
Carotene (mg g^−1^ f.w.)	0.21 ± 0.02 b	0.37 ± 0.03 a	0.31 ± 0.03 a
Total chlorophyll (mg g^−1^ f.w.)	3.99 ± 0.36 c	7.28 ± 0.66 b	9.12 ± 0.82 a
Chlorophyll a/b ratio	1.49	1.46	1.30
Chlorophyll/carotene ratio	19.0	19.7	29.4

Along each line, the values with the same letters do not differ statistically according to Duncan’s test at *p* < 0.05.

**Table 3:** Total antioxidant activity (AOA) and polyphenol (TP), proline, malonic dialdehyde (MDA) and lipid content in control and Se biofortified *A. annua* plants.

Parameter	Plant Part	Control	Se^+6^	Nano-Se
AOA (mg GAE g^−1^ d.w.)	Leaves	60.9 ± 6.0 a	61.7 ± 6.1 a	60.1 ± 5.9 a
	Stems	27.2 ± 2.6 a	30.3 ± 6.3 a	29.6 ± 2.0 a
	Roots	11.5 ± 0.6 a	11.8 ± 1.3 a	10.7 ± 1.0 a
TP (mg GAE g^−1^ d.w.)	Leaves	21.0 ± 1.2 a	21.4 ± 1.8 a	21.1 ± 2.0 a
	Stems	15.3 ± 1.4 a	16.8 ± 0.5 a	15.4 ± 1.3 a
	Roots	10.8 ± 0.6 a	11.1 ± 1.5 a	10.5 ± 1.0 a
Proline (mg g^−1^ d.w.)	Leaves	23.0 ± 2.1 b	18.0 ± 1.4 a	19.0 ± 1.7 a
Malonic dialdehyde (mg g^−1^ d.w.)	Leaves	9.2 ± 0.8 a	8.2 ± 0.7 a	7.6 ± 0.6 b
Wax (mg PE g^−1^ d.w.)	Leaves	7.60 ± 0.70 b	11.27 ± 1.00 a	9.70 ± 0.90 a

Along each line, the values with the same letters do not differ statistically according to Duncan’s test at *p* < 0.01.

**Table 4:** *A. annua* essential oil yield and composition of the control plants and plants supplied with sodium selenate and nano-Se.

Oil Component		Nano-Se		Se^+6^		Control	
		2022	2021	2022	2021	2022	2021
Essential oil yield (% d.w.)	M ± SD	0.35 ± 0.05 b	0.36 ± 0.04 b	0.75 ± 0.06 a	0.80 ± 0.06 a	0.75 ± 0.04 a	0.83 ± 0.07 a
	CV (%)	14.3	11.1	8.0	7.5	5.3	8.4
	Range	0.30–0.40	0.30–0.41	0.65–0.80	0.71–0.90	0.70–0.80	0.72–0.89
	Median	0.34	0.35	0.78	0.82	0.74	0.80
Eucalyptol		2.49 ± 0.23 d	2.11 ± 0.20 d	13.28 ± 1.10 a	10.85 ± 1.02 b	8.89 ± 0.81 c	15.58 ± 1.43 a
Artemisia ketone		3.97 ± 0.31 e	15.16 ± 1.32 c	10.20 ± 1.00 d	42.55 ± 4.12 a	13.36 ± 1.22 c	23.95 ± 2.17 b
Camphor		7.11 ± 0.70 d	5.01 ± 0.47 e	21.53 ± 2.02 b	13.78 ± 1.21 c	27.65 ± 2.16 a	25.89 ± 2.21 ab
Germacrene D		12.20 ± 1.11 a	2.40 ± 0.22 c	5.39 ± 5.11 b	0.93 ± 0.09 e	5.89 ± 0.53 b	1.45 ± 0.12 d
Total		25.77	24.68	50.40	68.11	55.79	66.85
N *		80	62	64	49	75	54

* number of components; along each line, the values with the same letters do not differ statistically according to Duncan’s test at *p* < 0.01.

**Table 5:** Polysaccharides, nitrates, total dissolved compounds (TDS) and wax content in the control and Se-fortified *A. annua* leaves.

Parameter	Control	Se^+6^	Nano-Se
Non-soluble Fiber (% d.w.)	50.4 ± 5.0 a	45.4 ± 4.5 a	47.4 ± 4.7 a
Soluble Fiber (% d.w.)	5.8 ± 0.5 a	6.0 ± 0.5 a	7.0 ± 0.6 a
Total Fiber (% d.w.)	56.2 ± 5.6 a	51.4 ± 5.1 a	54.4 ± 5.4 a
Pectin (% d.w.)	6.5 ± 0.6 c	11.0 ± 0.1 a	8.0 ± 0.8 b
Nitrates (mg g^−1^ d.w.)	3.12 ± 0.30 b	4.21 ± 0.40 a	3.97 ± 0.35 a
TDS (mg g^−1^ d.w.)	72.5 ± 7.1 b	88.9 ± 8.3 a	88.4 ± 8.4 a

Along each line, the values with the same letters do not differ statistically according to Duncan’s test at *p* < 0.01.

**Table 6:** Elemental composition of *A. annua* leaves as affected by sodium selenate and nano-Se (mg kg^−1^).

Element	Control	Se^+6^	Nano-Se
Macroelements			
Ca	6487 ± 649 c	8528 ± 850 b	13,885 ± 1380 a
K	29,229 ± 29,200 b	31,252 ± 3125 ab	36,636 ± 361 a
Mg	1861 ± 185 c	2517 ± 250 b	3592 ± 360 a
Na	230 ± 23 b	260 ± 26 b	446 ± 44 a
P	3887 ± 390 a	3319 ± 330 a	4069 ± 405 a
K/Na	127 ± 12 a	120 ± 12 a	82 ± 8 b
Microelements			
B	0.36 ± 0.03 c	0.45 ± 0.04 b	0.72 ± 0.07 a
Co	0.17 ± 0.02 b	0.09 ± 0.01 c	0.23 ± 0.02 a
Cu	13.12 ± 1.12 c	17.46 ± 1.52 b	21.44 ± 2.01 a
Fe	82.8 ± 8.3 c	112.0 ± 11.1 b	378.0 ± 37.1 a
I	1.05 ± 0.10 c	1.46 ± 0.13 b	3.20 ± 0.31 a
Li	0.22 ± 0.02 c	0.40 ± 0.04 b	0.77 ± 0.07 a
Mn	40.74 ± 4.0 b	45.16 ± 4.2 b	81.42 ± 8.0 a
Mo	0.42 ± 0.04 c	0.58 ± 0.05 b	0.71 ± 0.07 a
Se *	40 ± 3 c	2950 ± 285 a	980 ± 92 b
Si	3.16 ± 0.30 a	3.25 ± 0.30 a	3.24 ± 0.31 a
Zn	50.47 ± 5.0 b	44.92 ± 4.2 b	91.96 ± 9.1 a
As, Al and Heavy Metals			
Al	3.61 ± 0.33 b	3.49 ± 0.31 b	8.33 ± 0.80 a
As	0.08 ± 0.01 c	0.11 ± 0.01 b	0.32 ± 0.02 a
Cd	0.08 ± 0.01 b	0.09 ± 0.01 b	0.13 ± 0.01 a
Cr	0.29 ± 0.03 b	0.20 ± 0.02 c	0.75 ± 0.07 a
Ni	2.41 ± 0.23 a	2.43 ± 0.22 a	2.74 ± 0.25 a
Pb	0.17 ± 0.01 b	0.15 ± 0.01 b	0.40 ± 0.03 a
Sr	22.79 ± 2.3 c	31.65 ± 3.1 b	48.54 ± 4.8 a
V	0.11 ± 0.01 c	0.15 ± 0.01 b	0.71 ± 0.07 a

* In µg kg^−1^ d.w.; along each line, the values with the same letters do not differ statistically according to Duncan’s test at *p* < 0.05.

**Table 7:** Mean temperature and total rainfall during the experiment.

Month	2021		2022	
	Mean Temperature °C	Rainfall mm	Mean Temperature °C	Rainfall mm
April	9.6	41.3	11.8	41.4
May	16.3	20.2	14.9	24.5
June	19.9	175.2	22.9	83.5
July	26.3	59.5	24.3	22.3
August	25.1	97.6	26.0	20.4
September	17.9	50.0	19.6	12.1

## Data Availability

Not applicable.

## Sample Availability

Samples of the compounds are not available from the authors.
